# Do piperacillin/tazobactam and other antibiotics with inhibitory activity against *Clostridium difficile* reduce the risk for acquisition of *C. difficile* colonization?

**DOI:** 10.1186/s12879-016-1514-2

**Published:** 2016-04-18

**Authors:** Sirisha Kundrapu, Venkata C. K. Sunkesula, Lucy A. Jury, Jennifer L. Cadnum, Michelle M. Nerandzic, Jackson S. Musuuza, Ajay K. Sethi, Curtis J. Donskey

**Affiliations:** Department of Medicine, Infectious Diseases Division, Case Western Reserve, University School of Medicine, Cleveland, Ohio USA; Department of Pathology, University Hospitals Case Medical Center, Cleveland, Ohio USA; Geriatric Research Education and Clinical Center, Cleveland VA Medical Center, 10701 East Blvd, 44106 Cleveland, Ohio USA; Department of Population Health Sciences, University of Wisconsin School of Medicine and Public Health, Madison, Wisconsin USA

**Keywords:** *Clostridium difficile*, Colonization, Piperacillin/tazobactam

## Abstract

**Background:**

Systemic antibiotics vary widely in in vitro activity against *Clostridium difficile*. Some agents with activity against *C. difficile* (e.g., piperacillin/tazobactam) inhibit establishment of colonization in mice. We tested the hypothesis that piperacillin/tazobactam and other agents with activity against *C. difficile* achieve sufficient concentrations in the intestinal tract to inhibit colonization in patients.

**Methods:**

Point-prevalence culture surveys were conducted to compare the frequency of asymptomatic rectal carriage of toxigenic *C. difficile* among patients receiving piperacillin/tazobactam or other inhibitory antibiotics (e.g. ampicillin, linezolid, carbapenems) versus antibiotics lacking activity against *C. difficile* (e.g., cephalosporins, ciprofloxacin). For a subset of patients, in vitro inhibition of *C. difficile* (defined as a reduction in concentration after inoculation of vegetative *C. difficile* into fresh stool suspensions) was compared among antibiotic treatment groups.

**Results:**

Of 250 patients, 32 (13 %) were asymptomatic carriers of *C. difficile*. In comparison to patients receiving non-inhibitory antibiotics or prior antibiotics within 90 days, patients currently receiving piperacillin/tazobactam were less likely to be asymptomatic carriers (1/36, 3 versus 7/36, 19 and 15/69, 22 %, respectively; *P* = 0.024) and more likely to have fecal suspensions with in vitro inhibitory activity against *C. difficile* (20/28, 71 versus 3/11, 27 and 4/26, 15 %; *P* = 0.03). Patients receiving other inhibitory antibiotics were not less likely to be asymptomatic carriers than those receiving non-inhibitory antibiotics.

**Conclusions:**

Our findings suggest that piperacillin/tazobactam achieves sufficient concentrations in the intestinal tract to inhibit *C. difficile* colonization during therapy.

## Background

Antimicrobial exposure is the most important risk factor for *Clostridium difficile* infection (CDI) [1]. Clindamycin, third-generation cephalosporins, and fluoroquinolones are generally considered the agents that pose the greatest risk [1]. Restriction of these high-risk antibiotics may be a useful strategy to control CDI, but there is uncertainty regarding the selection of alternative agents that might have a lower propensity to cause CDI. Several recent studies have suggested that antibiotics with inhibitory activity against *C. difficile* (e.g., piperacillin/tazobactam, tigecycline, doxycycline, linezolid,) may pose a relatively low risk for CDI [2–7]. Moreover, Dubberke et al. [7] recently reported that cephalosporin use was associated with acquisition of *C. difficile* colonization, whereas β-lactam-β-lactamase inhibitor combinations and metronidazole use were associated with loss of colonization. Substitution of piperacillin/tazobactam for third-generation cephalosporins has been associated with reductions in the incidence of CDI [2, 3]. Similarly, substitution of piperacillin/tazobactam for cephalosporins has been associated with reductions in colonization with vancomycin-resistant enterococci (VRE) and extended-spectrum beta-lactamase-producing Enterobacteriacae [8, 9]. In mice, piperacillin/tazobactam and tigecycline inhibited acquisition of *C. difficile* colonization during treatment [10, 11]. However, it is not known if these agents achieve sufficient concentrations in the intestinal tract to inhibit *C. difficile* colonization in patients. In fact, Wilcox et al. [12] and Nord et al. [13] found significant inter-patient variability in excretion of piperacillin and tazobactam in stool of patients.

One primary objective of this study was to test the hypothesis that antibiotics with inhibitory activity against *C. difficile* are infrequently associated with *C. difficile* colonization during treatment in hospitalized patients. A second primary objective was to determine if antibiotics with in vitro inhibitory activity against *C. difficile* achieve sufficient concentrations in stool to inhibit in vitro growth of *C. difficile*. Our analysis focused primarily on piperacillin/tazobactam because it is the most commonly used broad-spectrum agent in our facility.

## Methods

### Ethics statement

The Cleveland VA Medical Center’s Institutional Review Board approved the study protocol. All subjects provided verbal informed consent for specimen collection and medical record review.

### Setting and participants

The Cleveland Veterans Affairs Medical Center is a 215-bed acute care hospital. During a 1-year period, intermittent point-prevalence culture surveys for asymptomatic carriage of toxigenic *C. difficile* were collected from consenting inpatients with no symptoms of CDI on 7 wards, including 3 medical wards, 1 surgical ward, a geriatric rehabilitation ward, a surgical intensive care unit, and a medical intensive care unit. The incidence of CDI was 7 cases per 10,000 patient-days during the study period with no outbreaks or clusters of CDI. Medical record review was conducted to obtain information on demographics, medical conditions, previous CDI, length of stay, antibiotic and subsequent development of CDI in the next 90 days. Based upon a modification of the classification scheme of Owens et al. [1], antibiotics on the formulary were classified as having inhibitory activity against *C. difficile* that was moderate to good (i.e., ampicillin, amoxicillin, linezolid, metronidazole, imipenem, meropenem, piperacillin/tazobactam, tigecycline, tetracyclines) or poor (i.e., cephalosporins, ciprofloxacin, and trimethoprim/sulfamethoxazole). Intravenous vancomycin was not classified as inhibitory because it achieves relatively low levels in the intestinal tract [14]. Moxifloxacin was classified as having variable activity against *C. difficile* because we have previously shown that isolates from our facility have variable susceptibility to moxifloxacin [15]. For patients receiving multiple antibiotics concurrently, the regimen was classified as inhibitory if one agent had moderate to good inhibitory activity.

### Microbiological methods

Peri-rectal swabs or fresh stool specimens were collected and transferred to an anaerobic chamber (Coy Laboratories, Grass Lake, MI). Swabs or aliquots of stool were plated directly onto pre-reduced *C. difficile* Brucella Agar containing taurocholic acid and lysozyme (CDBA-TAL) and incubated for 48 h [16]. In addition, to evaluate the possibility that carry over of antibiotics in stool to culture plates might result in false-negative cultures, all stool samples were serially diluted in phosphate-buffered saline to dilute any residual antibiotic and the dilutions were plated onto CDBA-TAL. Isolates were confirmed to be *C. difficile* on the basis of typical odor and appearance of colonies and by a positive reaction using *C. difficile* latex agglutination (Microgen Bioproducts, Camberly, UK). All *C. difficile* isolates were tested for in-vitro cytotoxin production using *C. difficile* Tox A/B II (Wampole Laboratories, Princeton, NJ), and isolates that did not produce toxin were excluded from the analysis.

### In vitro assay for inhibitory activity in fecal suspensions

To assess whether inhibitory concentrations of antibiotics were present in stool, a subset of patients whose fresh stool specimens were available after a minimum of 24 h of antibiotic treatment, were tested using a modification of the in vitro assay of colonization resistance developed by Borriello and Barclay [17]. To determine if inhibitory activity persisted after discontinuation of antibiotics, stool specimens collected up to 2 weeks after discontinuation of antibiotics were also analyzed. Up to 3 stool samples were analyzed per patient. The test strain was an epidemic NAP1/027/BI strain (VA 17). The minimum inhibitory concentrations (MICs) of several commonly used antibiotics for VA 17 are shown in Table 1. Fresh stool samples were homogenized in a 1:1 dilution of pre-reduced sterile water and inoculated with 10^4^ colony-forming units (CFU) per mL of vegetative *C. difficile* inside the anaerobic chamber. Serially-diluted samples were plated onto CDBA plates to determine the concentration of *C. difficile* immediately after inoculation and after incubation at 37 °C for 24 h. Samples with detectable levels of *C. difficile* prior to the inoculation of vegetative *C. difficile* were excluded. Specimens were considered inhibitory if the concentration of *C. difficile* decreased or remained unchanged compared to the baseline concentration. Inhibition of the *C. difficile* isolates was concurrently assessed in sterile filtrates of the stool suspensions. The filtrates were produced by centrifuging the suspensions at 10,000 rpm for 10 min, followed by filtering the supernatant through a 0.22 μm filter.

**Table 1:** Minimum Inhibitory Concentrations (MICs) of Commonly Used Antibiotics against the Clostridium difficile Test Strain

Antibiotic	MIC (μg/mL)^a^
Imipenem	2
Meropenem	2
Ampicillin	2
Piperacillin/tazobactam	2
Sulfamethoxazole/trimethoprim	32
Tetracycline	0.5
Ciprofloxacin	128
Moxifloxacin	64
Ceftriaxone	32
Clindamycin	>256
Metronidazole	0.25
Linezolid	0.5

^*a*^MICs were determined by broth dilution. The test strain (VA 17) is an epidemic NAP1/027/BI strain

### Statistical analysis

The frequency of asymptomatic carriage was compared for patients receiving piperacillin/tazobactam-containing or other inhibitory regimens versus those receiving non-inhibitory antibiotic regimens and/or prior antibiotics in the past 90 days. Log-binomial regression with robust variance estimation was used to model predictors of asymptomatic *C. difficile* carriage [18]. This approach yields a prevalence ratio with 95 % confidence intervals. Student *t*- and Wilcoxon rank sum tests were used for normally and non-normally distributed data, respectively. Fisher’s exact test was used to compare the proportions of stool suspensions that were inhibitory to growth of *C. difficile*. Data were analyzed with the use of SAS statistical software, version 9.1 (SAS Institute) and STATA 11 (StataCorp, College Station, TX).

## Results

### Frequency of and risk factors for asymptomatic carriage of *C. difficile*

Of 250 patients screened, 32 (13 %) were asymptomatic carriers of *C. difficile* and 218 were non-carriers. There was no evidence of clustering of colonized patients on specific wards (range, 2 to 7 colonized patients on each ward). As shown in Table 2, by bivariate analysis carriers were significantly more likely to have prior CDI or antibiotic treatment within 90 days. However, current antibiotic treatment was not associated with asymptomatic carriage. The length of stay at the time of culture collection was higher in those with asymptomatic carriage, but the difference was not statistically significant. Four of the asymptomatic carriers subsequently developed CDI, including 3 episodes of recurrent CDI and 1 initial episode.

**Table 2:** Characteristics of 32 Asymptomatic Carriers of *Clostridium difficile* and 218 Non-carriers

Characteristic	All participants (*N* = 250)	Asymptomatic carriers (*N* = 32)	Non-carriers (*N* = 218)	*P* value
Mean (SD) age (years)	66.9 (11.9)	64.3 (12.1)	67.2 (11.8)	0.19
Median (IQR) days from admission to culture	9 (4, 15)	11.5 (6, 20.5)	8 (4, 14)	0.07
Days from admission to culture				
0–7	110	13 (41)	97 (44)	0.34
8–14	75	7 (22)	68 (31)	
15–21	27	4 (12)	23 (11)	
≥22	38	8 (25)	30 (14)	
CDI in the previous 90 days	13	6 (19)	7 (3)	<0.001
Antibiotics in the past 90 days	155	27 (84)	128 (59)	0.005
Antibiotics in the past 30 days	144	24 (75)	120 (55)	0.03
Current antibiotic treatment	98	12 (38)	86 (39)	0.83
Comorbidities				
Heart disease	95	9 (28)	86 (39)	0.22
Cancer	85	12 (37)	73 (33)	0.65
Diabetes	103	11 (34)	92 (42)	0.40
End stage renal disease	23	3 (9)	20 (9)	0.97
Major surgery	70	8 (25)	62 (28)	0.69
Spinal cord injury	21	4 (13)	17 (8)	0.32

Data are no. (%) of patients, unless otherwise specified. *SD* standard deviation, *IQR* interquartile range

### Antibiotic therapy and *C. difficile* colonization

**Fig. 1:**
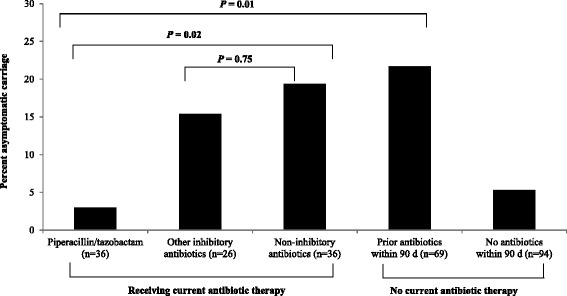
Frequency of asymptomatic carriage of toxigenic *Clostridium difficile* in hospitalized patients, stratified by antibiotic treatment classification. Based upon a modification of the classification scheme of Owens et al. [1], antibiotics on the formulary that were classified as having inhibitory activity against *C. difficile* included ampicillin, amoxicillin, linezolid, metronidazole, imipenem, meropenem, piperacillin/tazobactam, tigecycline, and tetracyclines. Antibiotics on the formulary that were considered to have poor activity included cephalosporins, ciprofloxacin, and trimethoprim/sulfamethoxazole. Moxifloxacin was classified as having variable activity against *C. difficile*

Figure 1 shows a comparison of the frequency of asymptomatic carriage of *C. difficile*, stratified by antibiotic treatment category. In comparison to patients receiving non-inhibitory antibiotics or prior antibiotics within 90 days, patients receiving current piperacillin/tazobactam were significantly less likely to be carriers of *C. difficile* (3 versus 18 and 22 %, respectively; *P* ≤ 0.02). Six of the 14 (43 %) patients with *C. difficile* carriage who had received prior antibiotics within 90 days had received regimens that included piperacillin/tazobactam. For the 6 patients who had received prior piperacillin/tazobactam, the mean number of days since the last dose of piperacillin/tazobactam was 7.5 (range, 2 to 14 days); for the 8 patients who received prior antibiotics other than piperacillin/tazobactam, the mean number of days since the last dose of antibiotics was 7.1 (range, 1 to 21 days). Patients receiving other inhibitory antibiotics were not less likely to be asymptomatic carriers than those receiving non-inhibitory antibiotics. Patients who had not received antibiotic therapy within the past 90 days were infrequently colonized with *C. difficile*.

Table 3 shows prevalence ratios of predictors of asymptomatic carriage generated using log-binomial regression analysis. Prior CDI and antibiotic use within 90 days were significant predictors of asymptomatic carriage, whereas current piperacillin/tazobactam use was protective in comparison to current non-inhibitory antibiotic use and/or antibiotic use in the past 90 days (*P* = 0.051).

**Table 3:** Prevalence Ratios of Predictors of Asymptomatic Carriage by Log-Binomial Regression Analysis

Characteristic	Prevalence ratio (asymptomatic carriage vs. non-carriage)	95 % CI	*P* value
Age (per year)	0.98	0.96, 1.01	0.18
Days from admission to culture (per day)			
0–7	1.00	Reference	
8–14	0.79	0.33, 1.89	0.6
15–21	1.25	0.44, 3.55	0.67
≥22	1.78	0.80, 3.97	0.16
CDI in the previous 90 days	4.21	2.11, 8.40	<0.001
Any antibiotics	0.93	0.48, 1.82	0.83
Antibiotics in the past 90 days	2.70	1.15, 6.33	0.02
Antibiotics in the past 30 days	2.20	1.03, 4.73	0.04
Inhibitory antibiotic activity (vs. non-inhibitory activity) against *C. difficile* (among antibiotic users)	0.41	0.14, 1.22	0.11
Piperacillin/tazobactam (vs. non-inhibitory antibiotics only)	0.14	0.02, 1.14	0.06
Piperacillin/tazobactam (vs. non-inhibitory and or antibiotics in past 90 days)	0.14	0.02, 0.99	0.05
Comorbidities			
Heart disease	0.64	0.31, 1.32	0.23
Cancer	1.16	0.60, 2.27	0.65
Diabetes	0.75	0.38, 1.48	0.41
End stage renal disease	1.02	0.34, 3.10	0.97
Major surgery	0.86	0.40, 1.82	0.69
Spinal cord injury	1.56	0.60, 4.03	0.36

*CI* Confidence interval

### In vitro inhibitory activity of fecal suspensions

**Fig. 2:**
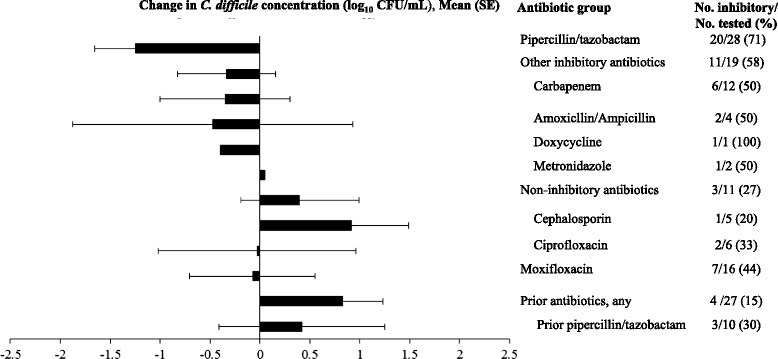
In vitro growth of *Clostridium difficile* in stool suspensions of hospitalized patients, stratified by level of in vitro inhibitory activity against *C. difficile* based on a modification of the classification of Owens et al. (1). A total of 141 stool specimens from 98 patients were analyzed (1–3 per patient). Specimens were considered inhibitory if the concentration of *C. difficile* decreased or remained unchanged compared to the baseline concentration. The non-inhibitory antibiotics that were administered at the time stool specimens were collected included ciprofloxacin (*N* = 6), ceftriaxone (*N* = 5), and cephalexin (*N* = 1). The agents classified as other inhibitory antibiotics that were administered included imipenem (*N* = 6), meropenem (*N* = 5), ertapenem (*N* = 6), linezolid (*N* = 4), ampicillin/sulbactam (*N* = 3), and metronidazole (*N* = 2). SE, standard error. CFU, colony-forming unit

Figure 2 provides a comparison of the proportions of fecal suspensions that were inhibitory to in vitro growth of *C. difficile* strain VA 17 and the mean (+/− standard error) change in *C. difficile* concentration, stratified by the type of antibiotic treatment. One-hundred forty-one stool specimens collected from 98 total patients were analyzed (1–3 per patient). In comparison to suspensions from patients on non-inhibitory antibiotics or with prior antibiotic exposure within 90 days, current piperacillin/tazobactam therapy was associated with more frequent inhibition of *C. difficile* growth (71 versus 27 and 44 %, respectively; *P* = 0.03). In contrast, only 30 % of stool suspensions from patients with prior rather than current piperacillin/tazobactam therapy were inhibitory to *C. difficile* growth; the time of collection of the specimens after discontinuation of piperacillin/tazobactam ranged from 1 to 7 days. Suspensions from patients on other inhibitory antibiotics were associated with more frequent inhibition in comparison to suspensions from patients with prior antibiotic exposure (*P* = 0.004), but not in comparison to suspensions from patients on non-inhibitory antibiotics (*P* = 0.14). Suspensions from patients on moxifloxacin were not associated with more frequent inhibition than suspensions from patients with prior antibiotic exposure or on non-inhibitory antibiotics (*P* = 0.07).

Of the 28 stool specimens analyzed during current piperacillin/tazobactam therapy, 10 (36 %) were from patients receiving this agent in combination with other antibiotics. There was no difference in the percentage of inhibitory specimens for piperacillin/tazobactam administered as monotherapy versus in combination with other antibiotics (7/10, 70 % versus 13/18, 72 %, respectively; *P* = 1). Of the 19 stool specimens analyzed during therapy with other inhibitory antibiotics, 7 (37 %) were from patients receiving these agents in combination with other antibiotics. There was no difference in the percentage of inhibitory specimens for these inhibitory agents administered as monotherapy versus in combination with other antibiotics (3/7, 43 % versus 8/12, 67 %, respectively; *P* = 0.38).

For fecal suspensions of patients receiving inhibitory antibiotics, fecal filtrates produced from inhibitory suspensions consistently inhibited growth of *C. difficile*, suggesting that inhibition was due to the presence of antibiotics rather than bacteria. In contrast, a majority of fecal filtrates produced from inhibitory suspensions of patients receiving non-inhibitory antibiotics did not inhibit growth of *C. difficile*.

## Discussion

Our findings suggest that piperacillin/tazobactam, an agent with activity against *C. difficile* that is excreted in significant concentrations in bile [10], achieves sufficient concentrations in the intestinal tract of many patients to inhibit *C. difficile* colonization during treatment. Patients receiving piperacillin/tazobactam therapy were infrequently colonized with *C. difficile* in comparison to patients currently receiving agents lacking activity against *C. difficile.* Moreover, 71 % of fecal suspensions or filtrates from patients currently receiving piperacillin/tazobactam were inhibitory to in vitro growth of *C. difficile*. These data are consistent with previous studies in mice and in an in vitro human gut model [10, 19]. However, given previous evidence of significant inter-patient variability in excretion of piperacillin and tazobactam in stool [12, 13], we cannot exclude the possibility that the infrequent colonization with *C. difficile* in piperacillin/tazobactam-treated patients may be attributable to low intestinal drug concentrations in some patients. In conjunction with previous studies, our results provide evidence that *C. difficile* colonization may be reduced by formulary substitution of piperacillin/tazobactam for cephalosporins.

We did not find that patients receiving other antibiotics with inhibitory activity against *C. difficile* (predominantly carbapenems and ampicillin or amoxicillin) were infrequently colonized with *C. difficile* in comparison to those receiving non-inhibitory antibiotics. This finding could potentially be related to low levels of biliary excretion of some agents (e.g., imipenem-cilastatin, meropenem) [20], or to inactivation in the intestinal tract (e.g., ampicillin may be inactivated in the colon by beta-lactamases) [21]. However, further studies are needed because the number of patients evaluated was limited, particularly for doxycycline and metronidazole.

We found relatively high rates of asymptomatic carriage of *C. difficile* in patients not on current antibiotics but who had received antibiotic therapy within the past 90 days. Moreover, only 15 % of stool samples tested from these patients were inhibitory to growth of *C. difficile*. These findings are consistent with recent studies that demonstrate that antibiotic therapy may result in prolonged disruption of the indigenous intestinal microbiota [22, 23]. Because piperacillin/tazobactam may cause disruption of the indigenous microbiota that persists after therapy, it should be appreciated that this agent may have a biphasic effect on colonization with *C. difficile* (i.e., inhibition during therapy but promotion after therapy during the period of recovery of the microbiota) [10]. Only 30 % of stool suspensions collected during the 2 week period after discontinuation of piperacillin/tazobactam were inhibitory to *C. difficile* and asymptomatic carriage was common after discontinuation of piperacillin/tazobactam (i.e., 43 % of carriers who had received prior antibiotics had received piperacillin/tazobactam).

Our study has several limitations. First, the study population included only men, a majority of whom were elderly. Additional studies are needed in other patient populations. Second, we collected a single rectal culture from many of the patients and therefore it is not known if the positive cultures represent persistent versus transient colonization. Third, our study may underestimate the true prevalence of asymptomatic carriage if many patients carry spores at levels below the limit of detection of our methods (~2 log_10_colony-forming units/gm of stool). Fourth, we did not examine susceptibility patterns of *C. difficile* specific to our institution. Fifth, as noted previously, more data is needed to evaluate the potential for agents such as doxycycline and linezolid to inhibit *C. difficile* colonization. Finally, we evaluated whether inhibitory antibiotics were associated with reduced asymptomatic carriage of *C. difficile*. Future studies are needed to determine whether inhibitory antibiotics reduce the risk for infection during therapy.

## Conclusions

Our findings demonstrate that piperacillin/tazobactam, an agent with activity against *C. difficile*, achieves sufficient concentrations in the intestinal tract of many patients to inhibit *C. difficile* colonization during treatment. These data suggest that *C. difficile* colonization may be reduced with the use of piperacillin/tazobactam compared to other regimens that are non-inhibitory to *C. difficile*. Further studies are needed to determine if formulary substitutions favoring use of piperacillin/tazobactam over cephalosporins might have an impact on CDI rates. Based on our findings, the potential impact of such formulary substitutions may be limited by the fact that piperacillin/tazobactam causes disruption of the indigenous microbiota that persists after therapy is discontinued.

### Ethics approval and consent to participate

The Cleveland VA Medical Center’s Institutional Review Board approved the study protocol. All subjects provided verbal informed consent for specimen collection and medical record review.

### Availability of data and materials

The data analyzed in this study can be accessed by sending a request to the corresponding author.

## Abbreviations

*C. difficile*: *Clostridium difficile*
CDBA-TAL: *C. difficile* Brucella Agar containing taurocholic acid and lysozyme
CDI: *Clostridium difficile* infection
CFU: colony-forming unit
SE: standard error

## References

[1] Owens RC, Donskey CJ, Gaynes RP, Loo VG, Muto CA (2008). Antimicrobial-associated risk factors for *Clostridium difficile* infection. Clin Infect Dis.

[2] Settle CD, Wilcox MH, Fawley WN, Corrado OJ, Hawkey PM (1998). Prospective study of the risk of *Clostridium difficile* diarrhea in elderly patients following treatment with cefotaxime or piperacillin-tazobactam. Aliment Pharmacol Ther.

[3] Wilcox MH, Freeman J, Fawley W, MacKinlay S, Brown A, Donaldson K (2004). Long-term surveillance of cefotaxime and piperacillin-tazobactam prescribing and incidence of *Clostridium difficile* diarrhea. J Antimicrob Chemother.

[4] Valerio M, Pedromingo M, Muñoz P, Alcalá L, Marin M, Peláez T (2012). Potential protective role of linezolid against *Clostridium difficile* infection. Int J Antimicrob Agents.

[5] Doernberg SB, Winston LG, Deck DH, Chambers HF (2012). Does doxycycline protect against development of *Clostridium difficile* infection?. Clin Infect Dis.

[6] Wilcox MH (2007). Evidence for low risk of *Clostridium difficile* infection associated with tigecycline. Clin Microbiol Infect.

[7] Dubberke ER, Reske KA, Seiler S, Hink T, Kwon JH, Burnham CA (2015). Risk factors for acquisition and loss of *Clostridium difficile* colonization in hospitalized patients. Antimicrob Agents Chemother.

[8] Quale J, Landman D, Saurina G, Atwood E, DiTore V, Patel K (1996). Manipulation of a hospital antimicrobial formulary to control an outbreak of vancomycin-resistant *enterococci*. Clin Infect Dis.

[9] Rice LB, Eckstein EC, DeVente J, Shlaes DM (1996). Ceftazidime-resistant *Klebsiella pneumoniae* isolates recovered at the Cleveland Department of Veterans Affairs Medical Center. Clin Infect Dis.

[10] Adams DA, Riggs M, Donskey CJ (2007). Effect of fluoroquinolone treatment on growth of and toxin production by epidemic and non-epidemic *Clostridium difficile* in the cecal contents of mice. Antimicrob Agents Chemother.

[11] Jump RL, Li Y, Pultz MJ, Kypriotakis G, Donskey CJ (2011). Tigecycline exhibits inhibitory activity against *Clostridium difficile* in the colon of mice and does not promote growth or toxin production. Antimicrob Agents Chemother.

[12] Wilcox MH, Brown A, Freeman J (2001). Faecal concentrations of piperacillin and tazobactam in elderly patients. J Antimicrob Chemother.

[13] Nord CE, Brismar B, Kasholm-Tengve B, Tunevall G (1993). Effect of piperacillin/tazobactam treatment on human bowel microflora. Antimicrob Chemother.

[14] Currie BP, Lemos-Filho L (2004). Evidence for biliary excretion of vancomycin into stool during intravenous therapy: potential implications for rectal colonization with vancomycin-resistant *enterococci*. Antimicrob Agents Chemother.

[15] Jump RL, Riggs MM, Sethi AK, Pultz MJ, Ellis-Reid T, Riebel W (2010). Multi-hospital outbreak of *Clostridium difficile*-associated disease, Cleveland, Ohio, USA. Emerg Infect Dis.

[16] Nerandzic MM, Donskey CJ (2009). An effective and reduced cost modified selective medium for isolation of *Clostridium difficile*. J Clin Microbiol.

[17] Borriello SP, Barclay FE (1986). An in-vitro model of colonisation resistance to *Clostridium difficile* infection. J Med Microbiol.

[18] Zou G (2004). A modified poisson regression approach to prospective studies with binary data. Am J Epidemiol.

[19] Baines SD, Freeman J, Wilcox MH (2005). Effects of piperacillin/tazobactam on *Clostridium difficile* growth and toxin production in a human gut model. J Antimicrob Chemother.

[20] Sullivan A, Edlund C, Nord CE (2001). Effect of antimicrobial agents on the ecological balance of human microflora. Lancet Infect Dis.

[21] Stiefel U, Pultz NJ, Harmoinen J, Koski P, Lindevall K, Helfand MS (2003). Oral administration of beta-lactamase preserves colonization resistance of piperacillin-treated mice. J Infect Dis.

[22] Abujamel T, Cadnum JL, Jury LA, Sunkesula VC, Kundrapu S, Jump RL (2013). Defining the vulnerable period for re-establishment of *Clostridium difficile* colonization after treatment of *C. difficile* infection with oral vancomycin or metronidazole. PLoS One.

[23] Hensgens MPM, Goorhuis A, Dekkers OM, Kuijper EJ (2012). Time interval of increased risk for *Clostridium difficile* infection after exposure to antibiotics. J Antimicrob Chemother.

